# Effects of gentle and rude vitality forms in social robots on humans during cognitive multitasking

**DOI:** 10.3389/frobt.2025.1305685

**Published:** 2025-04-22

**Authors:** Motonobu Aoki, Francesco Rea, Giuseppe Di Cesare, Giulio Sandini, Takura Yanagi, Atsushi Takamatsu, Tomohiro Yamamura

**Affiliations:** ^1^ Mobility and AI Laboratory, Nissan Motor Co., Ltd., Atsugi, Kanagawa, Japan; ^2^ Department of Robotics Brain and Cognitive Sciences, Italian Institute of Technology, Genoa, Liguria, Italy; ^3^ Department of Informatics, Bioengineering, Robotics and Systems Engineering, The University of Genoa, Genoa, Liguria, Italy; ^4^ Department of Medicine and Surgery, The University of Parma, Parma, Emilia–Romagna, Italy; ^5^ Research Planning Department, Nissan Motor Co., Ltd., Atsugi, Kanagawa, Japan

**Keywords:** human-robot interaction, social robots, vitality forms, cognitive multitasking, facial expressions, mental workload

## Abstract

Designing a social humanoid robot to enhance human cognitive multitasking in a mixed human-robot team is anything but straightforward. In fact, the robot’s presence and behavior can either improve or impair human performance. In this study, we examined how different vitality forms—expressed through the robot’s actions, speech and facial expressions—affect cognitive multitasking. Analysis of human facial expressions and skin conductance data revealed that a robot exhibiting a gentle vitality form fostered a more positive and relaxed state than one displaying a rude vitality form. Moreover, the gentle vitality form improved human performance on tasks involving short-term memory and continuous target tracking. To our knowledge, this is the first study to explore the long-term impact of vitality forms. Overall, our findings suggest that properly designing a social humanoid robot’s vitality forms can significantly enhance cognitive multitasking performance in human-robot teams.

## 1 Introduction

Humans communicate richly using explicit social signals such as verbal cues and with implicit social signals such as eye gaze (Argyle et al., 1973), tone of voice (Zuckerman et al., 1982), facial expressions (Zuckerman et al., 1982; Ekman, 1970), and gestures (Goldin-Meadow, 1999; Iverson and Goldin-Meadow, 1998). These social signals are integral to everyday interactions. Enabling robots to recognize and generate such social signals allows human-robot communication more effective and influencing human task performances. For example, Bartneck et al. showed that the mere presence of social robots can motivate people to put in more effort on a task (Bartneck, 2003) and Spatola et al. found that social robots improve performance on both the Stroop test and the Eliksen Flanker task (Spatola et al., 2019a; b). Moreover, Agrigoroaie and Tapus compared a robot that promoted impatience with one that remained motionless while encouraging a relaxed demeanor (Agrigoroaie and Tapus, 2020), finding that the former imposed a higher mental workload on participants, whereas the latter prompted them to relax, think carefully, and avoid undue worry. Rea et al. further reported that participants exercised harder and felt competitive with an impolite robot, while a polite robot was perceived as friendly, but sometimes uncompelling and disingenuous (Rea et al., 2021).

Although these studies have focused on the impact of social robots on single tasks, everyday life rarely involves only one task at a time. Instead people are often engaged in various form of collaborative multitasking—driving with a passenger, operating an aircraft with a co-pilot, or walking with a friend who offers advice and cautions each other while facing in the same direction. Therefore, in our previous study, we compared a robot that provided task advice through social signals during cognitive multitasking (Aoki et al., 2022) to evaluate social facilitation effects (Zajonc, 1965; Allport, 1924). The results indicated that while social robots worsen performance on a simple reactive task, it enhanced performance on a cognitively demanding task during multitasking. Additionally, our analysis of participants’ facial expressions revealed more positive expressions when they were with a socially behaving robot compared to a mechanically behaving one (Jirak et al., 2022).

Given the diversity of social behaviors, the different behavioral styles in social robots may affect human behaviors in various ways. For instance, Torrey et al. found that the use of hedges and discourse markers by robots can create a positive impression (Torrey et al., 2013) and Ghazali et al. showed that incorporating social cues reduces psychological reactance to highly controlling language (e.g., “you have to…”) (Ghazali et al., 2017). Building on our previous work in cognitive multitasking with social robots, this study focuses on how different behavioral styles—manifested through varying vitality forms—impact human performance.

## 2 Related work

### 2.1 Vitality forms in human-human interaction

Effective cooperation in social groups requires the ability to accurately predict others’ actions, interpret their behaviors, and adapt their activities. When interacting socially with others, we typically understand their behavioral goals and intentions (Brown and Brüne, 2012). This ability to attribute mental states—such as beliefs, intentions, knowledge—of others to ourselves and to predict their behavior based on these inferences is known as “theory of mind” (Premack and Woodruff, 1978). Evidence suggests that the fundamental mechanisms underlying theory of mind involve a group of neurons—neurons that fire both during action observation and execution (Gallese and Goldman, 1998; Rizzolatti and Craighero, 2004; Rizzolatti, 2005; Kilner et al., 2007).

During social interactions, people express their positive or negative attitudes through the way they perform actions or speak, using different vitality forms (e.g., gentle, rude). For instance, an agent may grasp and pass an object either rudely or gently, depending on their attitude. Variations in the force, direction, and velocity of their actions enable the receiver to infer their affective state and attitude. Daniel Stern defined these fundamental aspects of social communication as “vitality forms” (Stern, 2010). Vitality forms play a dyadic role in interpersonal relationships by enabling the agent to convey their attitudes and allowing the receiver to understand those attitudes (Di Cesare et al., 2017; 2020a; Rizzolatti et al., 2021).

Vitality forms are distinct from emotions. Scherer defines emotions as responses elicited by stimulus events—occurring when an event triggers a response in the organism after being appraised for its significance (Scherer, 2005). Furthermore, Pace-Schott et al. describe basic emotions as brief events characterized by visceromotor responses and behavioral preparation (Pace-Schott et al., 2019). In contrast, vitality forms represent human behaviors that reflect an agent’s internal emotional state (Stern, 2010). Specifically, social actions performed with a rude vitality form tend to exhibit larger trajectories and higher velocity profiles than those executed with a gentle vitality form (Di Cesare et al., 2016b). Additionally, speech delivered with a rude vitality form is characterized by a lower pitch (pitch of sound) and weaker intensity (volume) compared to speech delivered with a gentle vitality form (Di Cesare et al., 2017).

The ability to perceive and express vitality forms is evident even in infants during mother-infant interactions, underscoring their critical role in relating to and understanding others (Condon and Sander, 1974; Ammaniti and Ferrari, 2013; Rochat, 2009). Moreover, the perception of vitality forms is often impaired in individuals with social and communication deficits, such as children on the autism spectrum (Rochat et al., 2013). Most notably, Di Cesare et al. have studied the neural correlates of vitality form processing, showing that both the expression and perception of vitality forms activate the dorso-central insula and the middle cingulate cortex (Di Cesare et al., 2016a; Di Cesare et al., 2017; Di Cesare et al., 2020a; Di Cesare et al., 2021).

### 2.2 Vitality forms from social robots

While humans and monkeys both translate observed motor behaviors into their own internal motor representations, it has been shown that groups of neurons with mirror properties fire even when observing goal-directed actions performed by a different species (Ferrari et al., 2003). This finding indicates that mirror neurons in monkeys respond to human movement, suggesting they may play a role in understanding actions and intentions of others across species boundaries.

Additionally, Gazzola et al. identified the motor cortex involved in executing hand actions in humans and found that this area is strongly activated when observing actions performed by either humans or robots (Gazzola et al., 2007). Similarly, Oberman et al. demonstrated that mirror neurons are activated when observing hand movements by a humanoid robotic hand (Oberman et al., 2007). These studies indicated that mirror neurons encode the goal of an action, and that humans can project the apparent mental state of robots onto themselves. In a related experiment, Hegel et al. showed that people exert their theory of mind abilities on humanoid robots in a classical prisoner’s dilemma game (Hegel et al., 2008).

Furthermore, Di Cesare et al. argued that the same motor cortex is activated when observing a humanoid robot (iCub) (Metta et al., 2008; 2010) performing different vitality forms (Di Cesare et al., 2020b; Di Cesare, 2020; Vannucci et al., 2018). They proposed that differences in perceived vitality forms affect motor responses, with the same motor cortex firing during the observation of both human and robot actions. In their study, they implemented the kinematic features of human actions into the iCub robot, allowing it to exhibit vitality forms like a human. Most importantly, they demonstrated that observing these actions activated the brain network involved in processing vitality forms, just as it does when observing human actions (Di Cesare et al., 2020b). Notably, their results showed that the velocity profile and peak velocity are more crucial for representing vitality forms than adherence to the 2/3 power law.

Based on these studies, we aim to study how differences in the vitality forms of social robots impact human cognitive multitasking performance. The next section presents the study design.

## 3 Study design

This section outlines the hypothesis and experimental design, and tasks assigned to participants.

### 3.1 Hypotheses

Drawing on research into vitality forms and our previous studies on human cognitive tasks with social robots, we propose the following hypotheses:

• Hypothesis 1 (H1).
Participants complete a short-term memory task in less time in the presence of a gently behaving robot than in the presence of a rudely behaving robot.

• Hypothesis 2 (H2).
   Participants respond faster to a simple reactive task in the presence of a rudely behaving robot than in the presence of a gently behaving robot.

• Hypothesis 3 (H3).
    Participants control a target more accurately on a tracking task in the presence of a gently behaving robot than in the presence of a rudely behaving robot.

• Hypothesis 4 (H4).
Participants show more positive facial expressions and are more relaxed in the presence of a gently behaving robot than in the presence of a rudely behaving robot.

### 3.2 Experimental setup

**FIGURE 1:**
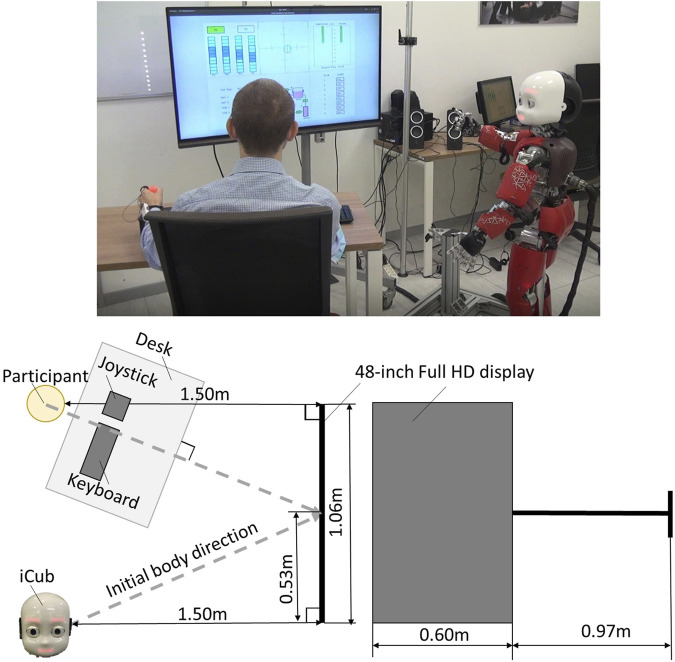
On the left is a plan view of the experimental layout, showing the positions of the participant, the iCub robot, the devices, and the display. On the right, is the dimensions of the display are shown.

**FIGURE 2:**
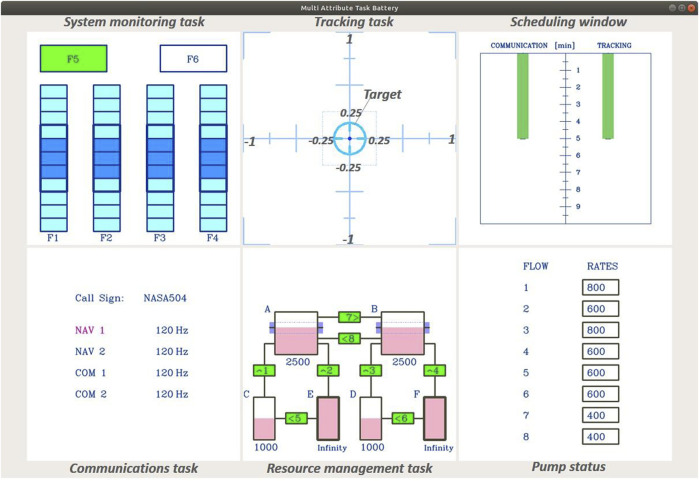
Display of the MATB-YARP task, composed of multiple modules.

All experiments are conducted with the humanoid robot iCub (Metta et al., 2008; Metta et al., 2010). The iCub design and control infrastructure enable it to reproduce human-like behaviors based on specific cognitive models of human-human interaction. Participants perform the MATB-YARP (see Section 3.3), a task battery that simulates various cognitive challenges encountered by aircraft pilots. Each participant engages with the MATB-YARP for 5 min. During this period, the iCub (see Section 3.4) marks the start and end of the task while continuously displaying vitality forms through coordinated movements of its arms, torso, and head. Figure 1 illustrates the experimental setup, and Figure 2 shows the MATB-YARP display.

### 3.3 The cognitive tasks (The MATB-YARP)

This study aims to explore human task performance and emotional states—as measured through facial expressions, gestures (including arm, torso, and head movements), gaze, and tone of voice—while participants engage in demanding cognitive multitasking in the presence of social robots exhibiting different behavioral styles based on vitality forms.

To this end, we selected MATB-YARP (Aoki et al., 2022), an implementation of NASA’s Multi-Attribute Task Battery (MATB) (Comstock Jr and Arnegard, 1992; Santiago-Espada et al., 2011) on YARP, which is suitable for experiments with the iCub. Among the task battery, we asked participants to perform the following three tasks for 5 min as in our previous study (Aoki et al., 2022).

#### 3.3.1 The communications task

**FIGURE 3:**
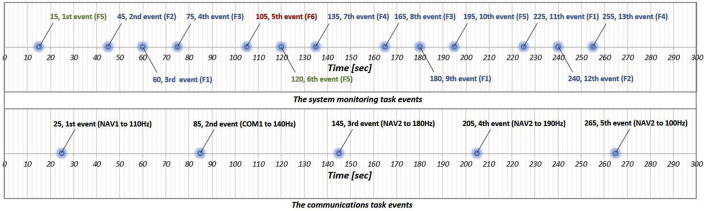
Overview of the MATB events for both the system monitoring task (first row) and the communications task (second row). Blue dots denote the timing of events that require input from participants.

The communications task is a task that requires participants to perform a series of key operations based on auditory information. In this task, participants adjust the radio frequencies of predetermined channels in response to voice instructions from a simulated air traffic control. Specifically, when instructed to adjust a radio frequency, they must select the appropriate channel (NAV1, NAV2, COM1, or COM2) using the *up* or *down* keys on a keyboard, navigate to the frequency change box with the *right* key, and then adjust the frequency using the *up* or *down* keys. The instructions is delivered in Italian, stating “*Change NAV1 (NAV2, COM1, COM2) to* X Hz”. During the task, participants can monitor the remaining duration of the communications task via a gauge on the left side of the scheduling window, which decreases as time passes. As shown in Figure 3, events in the communications task are designed to occur 5 times during 5-minute session. Overall, this task requires participants to briefly memorize the voice and then execute multiple operations accordingly.

#### 3.3.2 The system monitoring task

The system monitoring task is a simple reactive task designed to measure reaction time based on visual cues. It features four indicator lights and two warning lights. Participants must press the F5 key when the top-left box labeled “F5” changes from green to white and press the F6 key when the top-right box labeled “F6” changes from white to red. Pressing a key resets the corresponding box to its original color. Additionally, the four lower indicator lights—labeled “F1” through “F4”—move randomly within each default area indicated by each blue box while no event is occurring. If an indicator goes outside of its blue box, pressing the corresponding key returns it to the designated area indicated by the blue box. As shown in Figure 3, events in the system monitoring task are designed to occur 13 times in the 5-min session.

#### 3.3.3 The tracking task

The tracking task is a continuous, visually based compensatory task. In this task, participants use a joystick to keep a target centered. The target moves randomly in the x(−1.0to1.0) and y(−1.0to1.0) directions at the sampling rate of 30 Hz, following a Gaussian distribution ( σ = 0.008, μ = 0 in this study). The Gaussian parameter are designed so that the target can be maintained within the square when no other events are occurring, but it may be challenging to keep it inside when other task events take place. Participants are instructed to keep the target within a small central square (−0.25<x<0.25,−0.25<y<0.25) , and they can control its movement by tilting the joystick in the desired direction. The tracking task is executed continuously from the beginning to the end of the 5-minute period in which MATB-YARP is performed. Additionally, participants can monitor the remaining duration of the tracking task via a gauge on the right side of the scheduling window, which decreases as time passes. In this study, the average distance from the center is measured every 20 s from the start of the task.

### 3.4 Stimuli (robot behavior)

**FIGURE 4:**
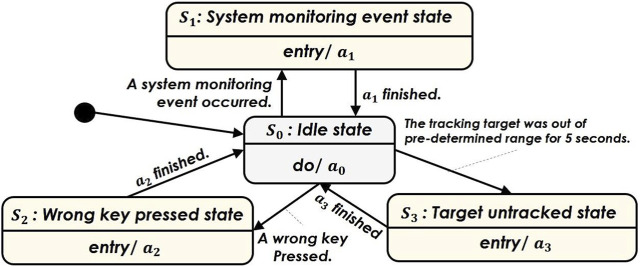
Finite state machine representing the flow of the interaction states with the iCub robot cf. Section 3.4.

In both the gentle and rude conditions, the iCub interacts with participants during the MATB-YARP exercise using physical movements and speech—all delivered in Italian. The two conditions differ in their vitality forms—that is, in the manner in which the robot behaves—which is detailed later in this section. The robot’s behavior in both conditions is governed by the finite state machine shown in Figure 4. Its control strategy transitions among four states: $S_{0}$ (Idle state), $S_{1}$ (System monitoring event state), $S_{2}$ (Wrong key pressed state), and $S_{3}$ (Target untracked state). These transitions are triggered by events within MATB-YARP and by specific inputs from participants during the 5-minute session. Among the three tasks in MATB-YARP, the robot supports participants through its behavior and speech during the tracking and system monitoring tasks. In contrast, the robot is designed to have no state transitions related to the communications task to prevent conflicts between its speech and the air traffic control guidance. Although the robot does not respond to the communications task, it is assumed that state transitions associated with the system monitoring, and tracking tasks may still impact performance on the communications task.

• $S_{0}$ : Idle state

  The robot monitors the task displayed on the screen. While monitoring, its arms, neck, and eyes moves continuously with the rhythm of human breathing.

• $S_{1}$ : System monitoring event state
When a system monitoring event is activated, the robot notifies the participant using pointing gestures toward the display, gazing into the participant’s face, and speech. The spoken phrase in Italian is: *“Press F1, please.”*

• $S_{2}$ : Wrong key pressed state
If the participant presses an incorrect key, the robot notifies the participant using pointing gestures toward the display, gazing into the participant’s face, and speech. The spoken phrase in Italian is: *“If I can help you, you have pressed the wrong key.”*

• $S_{3}$ : Target untracked state
When the participant fails to follow the tracking task, the robot alerts them using pointing gestures toward the display, gazing into the participant’s face, and speech. The spoken phrase in Italian is: “Sorry, you should correct your trajectory. Please pay attention.”

The speech content was recorded by an adult male actor, and its pitch was slightly raised to match the iCub’s appearance. These recordings were identical in both the gentle and rude conditions. In the following section, we outline the differences in between the two conditions.

#### 3.4.1 The gentle condition

**FIGURE 5:**
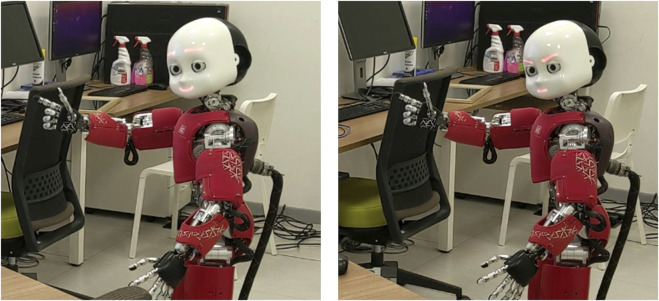
Robot behaviors and facial expressions in the gentle (the left image) and the rude (the right image) conditions.

In this condition, the robot exhibits a gentle vitality form characterized by calm, slow behaviors. Its movements are deliberately unhurried—each action, whether it is a pointing gesture, turning toward the participant, or reorienting its gaze to the monitor, lasts 3 s, which is slower than in the rude condition. When providing advice, the robot’s facial expression features level inner and outer eyebrows with slightly raised mouth corners (see Figure 5). Regarding voice, the robot uses weak plosives and accents, and it speaks slowly.

#### 3.4.2 The rude condition

In this condition, the robot exhibits the rude vitality form, characterized by aggressive and fast behaviors. Its movements are rapid—each action, whether it is a pointing gesture, turning toward the participant, or reorienting its gaze to the monitor, lasts 1.25 s, which is faster than in the gentle condition. When providing advice, the robot’s facial expression features raised outer eyebrows with mouth in a straight line (see Figure 5). Regarding voice, the robot uses a strong plosives and accents, and it speaks fast.

### 3.5 Integrated skin conductance response

To assess the participants’ mental workload during the multi tasks—i.e., to determine whether they are relaxed or not—we measure the electrodermal activity (EDA), also known as galvanic skin response (GSR), which is considered an indicator of sympathetic nervous system (Tao et al., 2019). EDA refers to changes in the skin electrical properties in response to sweat secretion. By applying a low constant voltage to the skin, we can noninvasively measure changes in skin conductance (SC) (Fowles et al., 1981).

The time series of SC can be divided into slow-changing tonic activity (skin conductance level, SCL) and rapid-changing phasic activity (skin conductance response; SCR).

In standard experimental settings, SCRs are often analyzed individually using peak detection methods. However, this approach assumes that responses are temporally isolated. In continuous tasks—such as those involving ongoing robot interactions—SCRs frequently occur in quick succession. As a result, subsequent responses may overlap with the decay phase of earlier ones, making it difficult to identify discrete peaks accurately. In such cases, peak detection methods tend to underestimate SCR amplitudes (Boucsein, 1992).

To address this issue, we adopt the integrated skin conductance response (ISCR) as our metric, following the approach proposed by Benedek and Kaernbach (Benedek and Kaernbach, 2010a; Benedek and Kaernbach, 2010b). ISCR is derived from the integral of the phasic driver and assumes that subsequent SCRs are additively superposed. This makes it particularly suitable for evaluating responses to continual stimuli, where traditional peak-based methods fall short.

Therefore, ISCR provides a more robust and accurate measure of mental workload under such conditions and is particularly well-suited to our study, which involves continuous task events and robot interactions that evoke overlapping SCRs. ISCR is defined by the following Equation 1:

$$ISCR=\int_{t_{1}}^{t_{2}}Driver_{\mathrm{phasic}}dt,$$ (1)

where $t_{1}$ and $t_{2}$ are the start and end times of the measurement, respectively. Furthermore, $Driver_{\mathrm{phasic}}$ is defined by the following Equation 2:

$$SC=\left(SC_{\mathrm{tonic}}+SC_{\mathrm{phasic}}\right)=\left(Driver_{\mathrm{tonic}}+Driver_{\mathrm{phasic}}\right)*IRF,$$ (2)

where IRF, impulse response function, is given by the Bateman function in Equation 3:

$$IRF=b\left(t\right)=e^{\frac{-t}{\tau_{1}}}-e^{\frac{-t}{\tau_{2}}},$$ (3)

with the optimal values for the time constants $\tau_{1}$ and $\tau_{2}$ calculated using the procedure described in the paper (Benedek and Kaernbach, 2010b).

For the calculation of $Driver_{\mathrm{phasic}}$ and ISCR, we use Ledapy 1.2.1 by Filetti (2020), a Python reimplementation of the MATLAB library Ledalab by Benedek and Kaernbach (2007). To assess the mental workload associated with each event, ISCR values are calculated every 20 s over the 5-minute task period. By examining the ISCR values at 20-second intervals, we determine the mental workload during each time segment. As an EDA sensor, we employ a Shimmer3 GSR + module, which has been proven to be a reliable and accurate wearable sensor platform for recording biological signals (Burns et al., 2010b; Burns et al., 2010a). In our experiment, its electrodes are attached to the index and middle fingers of the participants’ left hands.

### 3.6 Arousal and valence from facial expressions

In this study, participants’ facial expressions are recorded, and arousal and valence (Russell, 1980) are estimated to determine whether the robot’s vitality forms during the task propagate to the participants. According to one approach, all emotional states arise from two basic neurophysiological systems—arousal and valence—a concept known as the “circumplex model of affect” (Posner et al., 2005). Higher arousal indicates that the participant is less drowsy and more energetic, while higher valence signifies a more positive emotional state. Facial expressions are continuously recorded throughout the 5-minute tasks using a USB camera mounted on the monitor displaying MATB-YARP, capturing images at approximately 12–15 FPS. For each frame, arousal and valence are estimated using a deep learning model. Specially, we adopted the FaceChannel deep neural network architecture (Barros et al., 2020), which was initially trained on the FER + dataset (Barsoum et al., 2016) and fine-tuned on the AffectNet dataset (Mollahosseini et al., 2017). In the FaceChannel network, both arousal and valence are represented on a scale ranging from −1 to 1. By calculating the mean values over 20-second intervals, we obtain the arousal and valence values for each time period.

### 3.7 Procedure

Participants are welcomed in a room arranged to be comfortable and distinctly different from a typical laboratory setting. To minimize distractions that might affect performance, the experimenter’s control area is placed behind the participants. The robot begins in its home position (expressionless, standing, and facing forward), and as soon as the participant enters the room, the following protocol is implemented:

1. Participants are asked to sit in the designated chair in the experimental setup.
2. Before the experiment begins, the experimenters explain how to operate the MATB-YARP task and instruct participants to wear the EDA sensor on their left hand. They are also directed to use the joystick with their left hand and the keyboard with their right hand.
3. The robot signals the start of the MATB-YARP task by speaking in Italian: “Are you ready to start? I have to inform you that the experiment is starting, let’s start the experiment, I am here for you.” The task then commences, with events occurring as scheduled in the time series. The robot’s behavior speed and its tone of voice vary depending on the condition.
4. After 5 min, the tasks end. The robot indicates the conclusion of the MATB-YARP task by speaking in Italian: “Thank you, the experiment is terminated.” Again, the robot’s behavior speed and its tone of voice vary depending on the condition.
5. Finally, the experimenter conducts a post-experiment interview to verify that the participant has observed the iCub’s behaviors.

The experimental protocol is designed to minimize interaction between participants and the experimenters, ensuring that the entire experience focuses on the interaction between the humanoid robot and the participant.

### 3.8 Participants

We recruited 29 native Italian speakers (18–54 years old, M = 32.5, SD = 153.6) from citizens in Genoa, Italy and randomly assigned 15 (9 female, 6 male) to the gentle condition and 14 (9 female, 5 male) to the rude condition to mitigate recruitment bias. All participants volunteered for the study and received no financial compensation. None were familiar with the iCub robot, and all had completed at least an undergraduate degree. Each participant signed an informed consent form approved by the IIT ethical committee. Participants also consented to use of camera and microphone recordings during the experiment and to the use of their data for scientific purposes. The research adhered to the ethical standards in the 1964 Declaration of Helsinki and was approved by the local ethical committee of the Liguria Region in Italy (n. 222REG2015), which protects participants.

### 3.9 Analysis

We recorded data from 15 participants (9 female, 6 male) of the gentle condition and 15 participants (9 female, 6 male) of the rude condition. Data from one participant in the rude condition was excluded because the recording was stopped mid-session, so the analysis for that condition included data from the remaining 14 participants (9 female, 5 male).

For data analysis, we assessed the following behavioral measures in the tasks:

• The communications task: Time from each event to the completion of the radio frequency adjustment.

• The system monitoring task: Reaction time from each event.

• The tracking task: Target distance from the center.

In the communications task, events that take longer than 15 s to complete—or that receive no response—were assigned a completion time of 15 s. In our experiment, 17 responses (5 in the gentle condition and 12 in the rude condition) were treated this manner, resulting in a total of 145 responses (75 in the gentle condition and 70 in the rude condition) included in the analysis.

In the system monitoring task, events that take longer than 5 s to react to—or that receive no response—were assigned a reaction time of 5 s. In our experiment, 40 responses (22 in the gentle condition and 18 in the rude condition) were treated in this manner, resulting in a total of 377 responses (195 in the gentle condition and 182 in the rude condition) included in the analysis.

In the tracking task, the target’s distance from the center was averaged every 20 s, yielding 15 data points per participant. In our experiment, 435 data points were obtained from 29 participants (225 from 15 participants in the gentle condition and 210 from 14 participants in the rude condition) and included in the analysis.

For the facial expression data, we averaged the arousal and valence values every 20 s, yielding 15 data points per participant. Due to missing data, we excluded 1 participant from the gentle condition and 3 participants from the rude condition, leaving us with 375 data points from 25 participants (210 from 14 participants in the gentle condition and 165 from 11 participants in the rude condition).

In addition, the ISCR was calculated every 20 s during the 5-minute task, yielding 15 data points per participant. If a participant’s mean ISCR exceeded 10 μSs (i.e., the mean SCR was more than 0.5 μS), we considered that they experience excessive mental workload due to unfamiliarity with the experiment and excluded their data. Similarly, if a participant’s mean ISCR fell below 0.2 µSs (i.e., the mean SCR was less than 0.01 µS), we considered it unlikely that they exhibited normal skin conductance responses, and we excluded their data. In our experiment, we excluded one participant from the gentle condition and three from the rude condition on this basis, leaving 375 data points from 25 participants (210 from 14 participants in the gentle condition and 165 from 11 participants in the rude condition).

For statistical analysis, we applied the Mann-Whitney U test (Mann and Whitney, 1947; Wilcoxon, 1992) to the performances of the system monitoring task, the communications task, the tracking task, as well as arousal, valance, and ISCR. We used R 4.1.2 for these tests.

We also compared the values for each condition to baseline values, which were obtained from another group of 14 participants (5 female, 9 male) who performed the MATB-YARP task without any robot present. The baseline values were calculated as median values of each task performance, arousal, valence, and ISCR. We then examined the differences between the median values in the gentle and rude conditions, as well as their respective baseline values.

## 4 Results

In this section, we summarize the experimental results in relation to the four hypotheses (see Section 3.1), our results support H1, H3, and H4 but not H2. A summary of the data is provided in Table 1.

**TABLE 1:** The statistical values obtained in this study are summarized.

Items	Number of data		Median		U	PS	95%CI		p	
	gentle	rude	gentle	rude			lower	upper		
Sysmon [s]	195	182	2.11	2.11	1.7e+04	0.49	−0.33	0.26	0.76	
Comm [s]	75	70	6.88	7.95	2.1e+03	0.40	−3.67	−0.060	0.040	*
Tracking	225	210	0.10	0.12	1.6e+04	0.35	−0.056	−0.027	6.9e-08	****
Arousal	225	210	0.010	−0.0052	1.7e+04	0.49	−0.053	−0.038	0.64	
Valence	225	210	0.049	−0.023	1.5e+04	0.57	0.023	0.25	0.019	*
ISCR [ μ Ss]	210	165	0.79	1.31	1.5e+04	0.43	−0.998	−0.082	0.0014	**

Here, “Sysmon”, “Comm”, and “Tracking” represent the results of the system monitoring, the communications, and the tracking tasks, respectively. “ U ” denotes the Mann-Whitney U test statics, “PS” indicates the probabilities of superiority, and “ p ” represents the p-values. (∗=p<0.05,∗∗=p<0.01,∗∗∗=p<0.001,∗∗∗∗=p<0.0001.)

### 4.1 Hypothesis 1: supported

**FIGURE 6:**
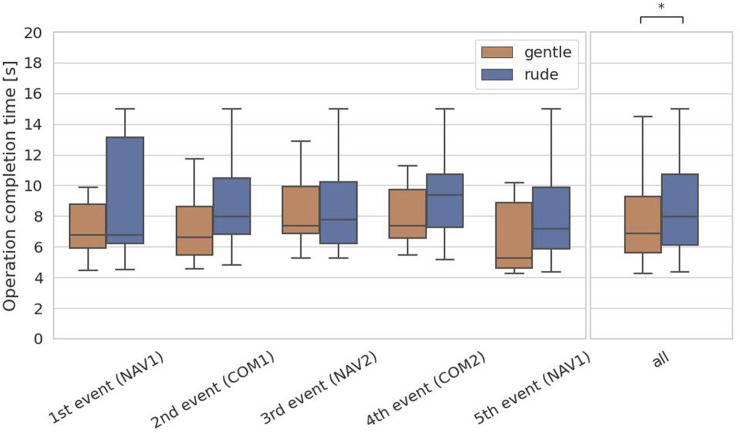
Evaluation of the operation completion time on each event and at all events in the communications task. (∗ = *p* < 0.05, ∗ ∗ = *p* < 0.01, ∗ ∗ ∗ = *p* < 0.001.)

The first hypothesis examined whether the presence of a gently behaving robot significantly enhances on human performance in a cognitive task requiring short-term memory, compared to a rudely behaving robot. To test the hypothesis, we evaluated the operation completion time of the communication task, which is associated with short-term memory. For each participant, we obtained 5 data samples, and we applied the Mann-Whitney U test to the responses to the communications task events. As shown in Figure 6, the operation completion time in the gentle condition was significantly shorter than in the rude condition $\left(p=0.040,U=2.1\times 10^{3}\right)$ . This result supports hypothesis 1.

### 4.2 Hypothesis 2: not supported

**FIGURE 7:**
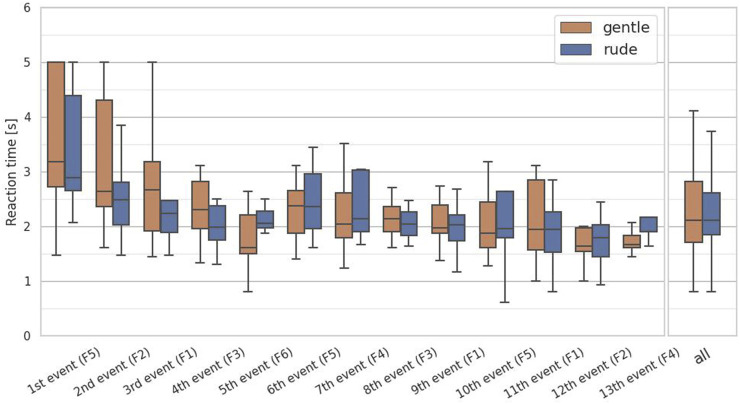
Evaluation of the reaction time on each event and at all events in the system monitoring task. (∗ = *p* < 0.05, ∗ ∗ = *p* < 0.01, ∗ ∗ ∗ = *p* < 0.001.)

The second hypothesis examined whether the presence of a rudely behaving robot significantly affect human reactive performance on a cognitive task, compared to a gently behaving robot. To test the hypothesis, we analyzed the reaction time in the system monitoring task. For this task, we collected 13 data samples per participant and applied the Mann-Whitney U test to the responses. As shown in Figure 7, there was no significant difference in the reaction times $\left(p=0.76,U=1.7\times 10^{4}\right)$ , and thus hypothesis 2 is not supported.

### 4.3 Hypothesis 3: supported

**FIGURE 8:**
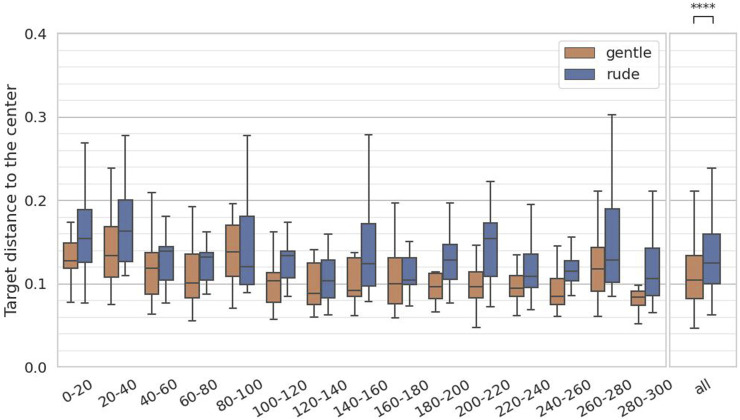
Evaluation of the target distance to the center on every 20 s and at all periods in the tracking task. (∗ = *p* < 0.05, ∗ ∗ = *p* < 0.01, ∗ ∗ ∗ = *p* < 0.001.)

The third hypothesis examined whether the presence of a gently behaving robot significantly improve on human tracking performance in a cognitive task requiring continuous focus, compared to a rudely behaving robot. To test the hypothesis, we measured the distance from the center in the target in the tracking task. We calculated the average distance to the center at 20-second intervals, yielding 15 data samples per participant, and we applied the Mann-Whitney U test. As shown in Figure 8, the distance in the gentle condition was significantly smaller than in the rude condition $\left(p=6.9\times 10^{-8},U=1.6\times 10^{4}\right)$ . Therefore, hypothesis 3 is supported.

### 4.4 Hypothesis 4: supported

**FIGURE 9:**
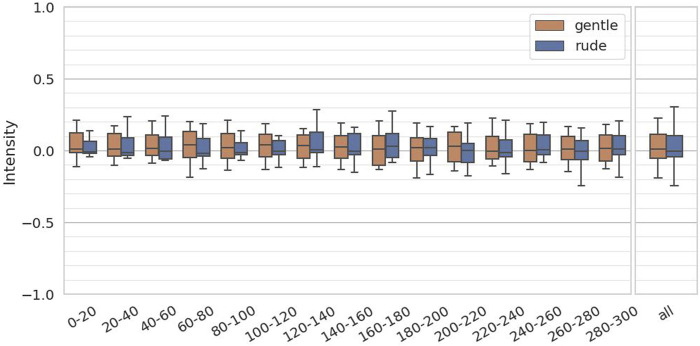
Evaluation of the arousal values on every 20 s and at all periods. (∗ = *p* < 0.05, ∗ ∗ = *p* < 0.01, ∗ ∗ ∗ = *p* < 0.001.)

**FIGURE 10:**
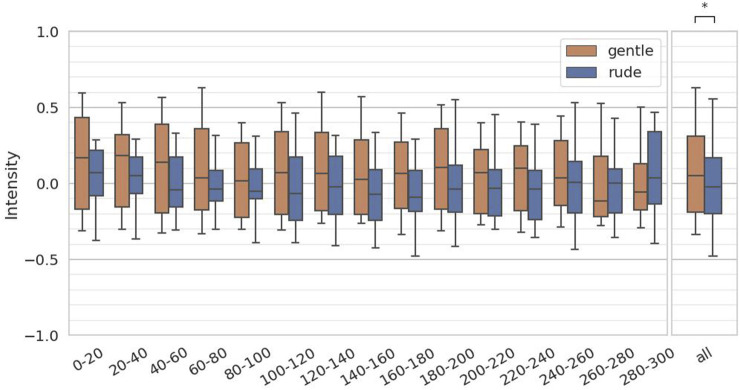
Evaluation of the valence values on every 20 s and at all periods. (∗ = *p* < 0.05, ∗ ∗ = *p* < 0.01, ∗ ∗ ∗ = *p* < 0.001.)

**FIGURE 11:**
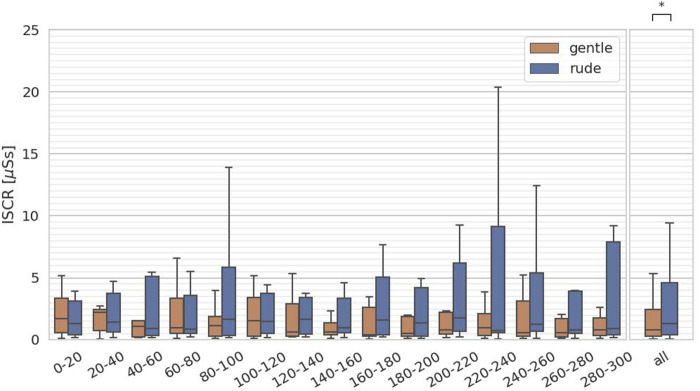
Evaluation of the integrated skin conductance response (ISCR) on every 20 s and at all periods. (∗ = *p* < 0.05, ∗ ∗ = *p* < 0.01, ∗ ∗ ∗ = *p* < 0.001.)

The fourth hypothesis examined whether the presence of a gently behaving robot significantly affects human facial expressions and mental workload, compared to a rudely behaving robot. To test this, we evaluated arousal and valence from facial expressions, as estimated by the FaceChannel deep neural network, and the ISCR from the skin conductance measurements. For both arousal and valence, we computed the mean values over 20-second intervals, yielding 15 data samples per participant, and we applied the Mann-Whitney U test. As shown in Figure 9, there was no statistically significant difference in arousal between the conditions $\left(p=0.64,U=1.7\times 10^{4}\right)$ . However, as shown in Figure 10, valence is significantly higher in the gentle condition than in the rude condition $\left(p=0.019,U=1.9\times 10^{4}\right)$ . Similarly, ISCR values—calculated every 20 s (15 samples per participant) and analyzed with the Mann-Whitney U test—are significantly lower in the gentle condition than in the rude condition $\left(p=0.0014,U=1.5\times 10^{4}\right)$ as shown in Figure 11. These results indicate that participants exhibited more relaxed and positive facial expressions in the gentle condition. Therefore, hypothesis 4 is supported.

### 4.5 Comparison with the baseline

We compared the median values in each condition against the baseline, as summarized in Table 2. For the system monitoring task, reaction times were 0.24 s slower than baseline in both the gentle and rude conditions. In the communications task, the operation completion time was 0.02 s slower than baseline in the gentle condition and 1.09 s slower in the rude condition. In the tracking task, performance was 0.012 units worse than baseline in the gentle condition and 0.032 units worse in the rude condition. For arousal, values were 0.014 higher than baseline in the gentle condition and 0.001 lower in the rude condition. For valence, values were 0.167 higher than baseline in the gentle condition and 0.095 higher in the rude condition. Finally, for ISCR, values were 0.565 μ Ss higher than baseline in the gentle condition and 1.085 μ Ss higher in the rude condition.

**TABLE 2:** The differences between the values obtained in this study and the baseline values are summarized. The baseline was measured when the MATB-YARP task was performed without any robot present.

Items	Median				
	baseline (alone)	gentle	rude	gentle-baseline	rude-baseline
SysMon [s]	1.87	2.11	2.11	0.24	0.24
Comm [s]	6.86	6.88	7.95	0.02	1.09
Tracking	0.088	0.10	0.12	0.012	0.032
Arousal	−0.004	0.010	−0.005	0.014	−0.001
Valence	−0.118	0.049	−0.023	0.167	0.095
ISCR [ μ Ss]	0.225	0.790	1.310	0.565	1.085

## 5 Discussion

We examined the effects of two interaction styles of humanoid robots on multitasking performance, facial expressions, and mental workload. Data were collected from 29 participants during a multitasking experiment in which the robot provided advice for the system monitoring and tracking tasks—but not for the communications task. Our results showed that participants in the gentle condition performed better in the communications task—which required short-term memory of voice instructions and multiple key presses—and in the tracking task, which demanded continuous attention.

Moreover, analysis of valence and ISCR derived from facial expressions revealed that participants exhibited more positive emotion and lower unconscious mental workload when being with the gently moving robot. These findings suggest that the gentle behavior with the cooperative expressions of the robot unconsciously fostered a positive, relaxed state in the participants, thereby improving their performance on the communications task and demonstrating the influence of vitality forms.

Conversely, in the system monitoring task—a simple reactive task—there was no statistically significant difference between the two conditions, and the influence of vitality forms was not evident. In a previous study by Di Cesare et al. (2020b), participants received an object after observing an iCub offering it either gently or rudely. In our experiment, however, participants merely pressed keys on a keyboard, so any differences in reactive movement speed were likely less pronounced.

We also observed that the likelihood of failing to complete the communications task within 15 s was lower in the gentle condition (5 out of 75 responses) compared to the rude condition (12 out of 70 responses). This suggests that rude robot behavior imposed a higher mental workload, causing participants to take longer in their decision-making process.

We further compared the median values in each condition to baseline values obtained when the MATB-YARP task was performed without any robot present. As noted in Section 4.5, reaction times in the system monitoring task were equally slower in both the gentle and rude conditions compared to the baseline. This suggests that for simple reactive tasks, it may be preferable to work alone to minimize distractions. In contrast, for the communications task—a cognitively demanding task—performance in the gentle condition was nearly identical to baseline, while performance in the rude condition deteriorated. Similarly, in the tracking task, which requires sustained concentration, the rude condition negatively impacted performance. Additionally, ISCR values were higher than baseline in both conditions, with the rude condition showing an even greater increase.

These results suggest that merely the social and physical presence of a robot can elevate mental workload, and this effect is amplified when the robot behaves rudely. Notably, valence values were even higher in the gentle condition—exceeding those in both the rude condition and the baseline—indicating that participants experienced more positive emotions when interacting with a gentle robot. The fact that the rude condition also yielded slightly higher valence values than the baseline implies that simply interacting with a social robot for the first time may have a modestly positive emotional effect.

Taken together, a gentle social robot does not increase mental workload as much as a rude robot; rather, it elicits more positive emotions and reduces the likelihood of errors. These findings suggest that the presence of a gentle robot has the potential to enhance team performance in cognitive multitasking. Future research should explore these effects over longer periods to better understand the impact of interactions with robotic agents.

## 6 Limitation

This study has several limitations.

While further research is needed to reinforce our conclusions, some findings are clearly evident. A review of camera images shows that all participants observed the iCub’s behavior at the beginning and end of the 5-minute session. In contrast, only two participants in the gentle condition and none in the rude condition looked at the iCub during the tasks—with those participants glancing only two or three times, respectively. Nevertheless, post-experiment interviews confirmed that all participants were aware of the iCub’s gesturing during the tasks, as the robot was positioned within their peripheral view. Thus, it is likely that while participants recognized some of the robot’s behaviors, they did not observe all of them, and some of the participants were not influenced by these behaviors. Given these facts, additional study is needed to further divide the experimental conditions into voice-only and behavior-only to clarify the effects of each component.

Another limitation is that our between-subjects design prevented us from asking participants whether they could distinguish between the “gentle” and “rude” vitality forms. Subsequent physiological experiments could then measure the activity of the brain networks involved in processing vitality forms, providing direct evidence that they were indeed propagated to the participants.

Also due to the fact that the sample size was not so large, it was not sufficient to develop a predictive model combining each of the parameters. If more data had been collected, a more informative statistical analysis could have been performed.

Lastly, this study involved participants performing the three MATB tasks simultaneously. Future studies might enhance our understanding by having participants perform each individual task separately.

## 7 Conclusion

This paper contributed to the burgeoning field of vitality forms research, which is attracting increasing attention in neuroscience. Unlike previous experiments on vitality forms that measured a single human reaction to one isolated robot action, our research evaluated participants’ performance over a 5-minute period during a series of robot behaviors. This approach provided a more comprehensive view of the impact of vitality forms over an extended timeframe. Furthermore, these results may serve as a stepping stone for exploring the long-term effects of vitality forms in real-world settings beyond robotics. Overall, our findings suggest that properly designing a social humanoid robot’s vitality forms can significantly enhance cognitive multitasking performance in human-robot teams.

## Data Availability

The raw data supporting the conclusions of this article will be made available by the authors, without undue reservation.
